# Cryptococcal meningoencephalitis with *Actinomyces odontolyticus* sepsis: a case report and literature review

**DOI:** 10.1186/s12879-023-08391-w

**Published:** 2023-06-27

**Authors:** Qingyu Huang, Zhuquan Hong, Quanlong Hong

**Affiliations:** Department of Neurology, Quanzhou First Hospital Affiliated to Fujian Medical University, Quanzhou, 362000 Fujian China

**Keywords:** *Actinomyces Odontolyticus* Sepsis, *Cryptococcus Neoformans* Meningoencephalitis, Combined Infection

## Abstract

**Background:**

The combined infection of *actinomyces odontolyticus* sepsis and cryptococcal encephalitis is rare in routine clinical practice. Thus, we presented this case report and literature review to provide clues to improve such patients' diagnoses and treatment processes.

**Case presentation:**

The main clinical manifestations of the patient were high fever and intracranial hypertension. Then, we completed the routine cerebrospinal fluid examination, biochemical detection, cytological examination, bacterial culture, and India ink staining. Firstly, the blood culture suggested *actinomyces odontolyticus infection*, considering the possibility of *actinomyces odontolyticus* sepsis and intracranial *actinomyces odontolyticus* infection. Accordingly, the patient was administered penicillin for treatment. Although the fever was slightly relieved, the symptoms of intracranial hypertension did not relieve. After 7 days, the characteristics of brain magnetic resonance imaging and the results of pathogenic metagenomics sequencing and cryptococcal capsular polysaccharide antigen suggested that cryptococcal infection. Based on the above results, the patient was diagnosed with a combined infection of cryptococcal meningoencephalitis and actinomyces odontolyticus sepsis. Anti-infection therapy with ‘penicillin, amphotericin, and fluconazole’ was provided, improving the clinical manifestations and objective indexes.

**Conclusion:**

The combined infection of *Actinomyces odontolyticus* sepsis and cryptococcal encephalitis is first reported in this case report, and combined antibiotics with ‘penicillin, amphotericin, and fluconazole’ are effective.

## Background

Cryptococcal meningoencephalitis in patients without HIV infection is fungal meningitis with various clinical manifestations. Although actinomycosis is a chronic granulomatous disease with slow progression, it may cause endogenous infection when tissue damage or inflammation causes hypoxia and a decrease in local resistance. The combined infection of *Actinomyces odontolyticus* sepsis and cryptococcal encephalitis is rare in routine clinical practice. The present study reported a case of a patient with cryptococcal meningoencephalitis with *Actinomyces odontolyticus* sepsis who was admitted to our hospital. It is expected to improve this disease's clinical diagnosis and treatment through a case report and relevant literature review.

## Case presentation

A 50-year-old male patient was transferred to our hospital with a chief complaint of ‘repeated head and neck discomfort for over 1 month and aggravation for 5 days’. In detail, the patient showed head and neck tightness and discomfort more than 1 month before, accompanied by progressive persistent oppressive pain of the whole head. Five days ago, the patient developed an aggravated headache, accompanied by nausea and vomiting several times, abnormal behavior (mistaking night as early morning), vague speech, weak voice, and unstable walking. The patient had an intermittent occurrence of ‘caries and broken teeth’ 2–3 years ago and showed ‘stroke and hypertension’ 1 year ago. In addition, the patient raised chickens and ducks at home.

**Fig. 1:**
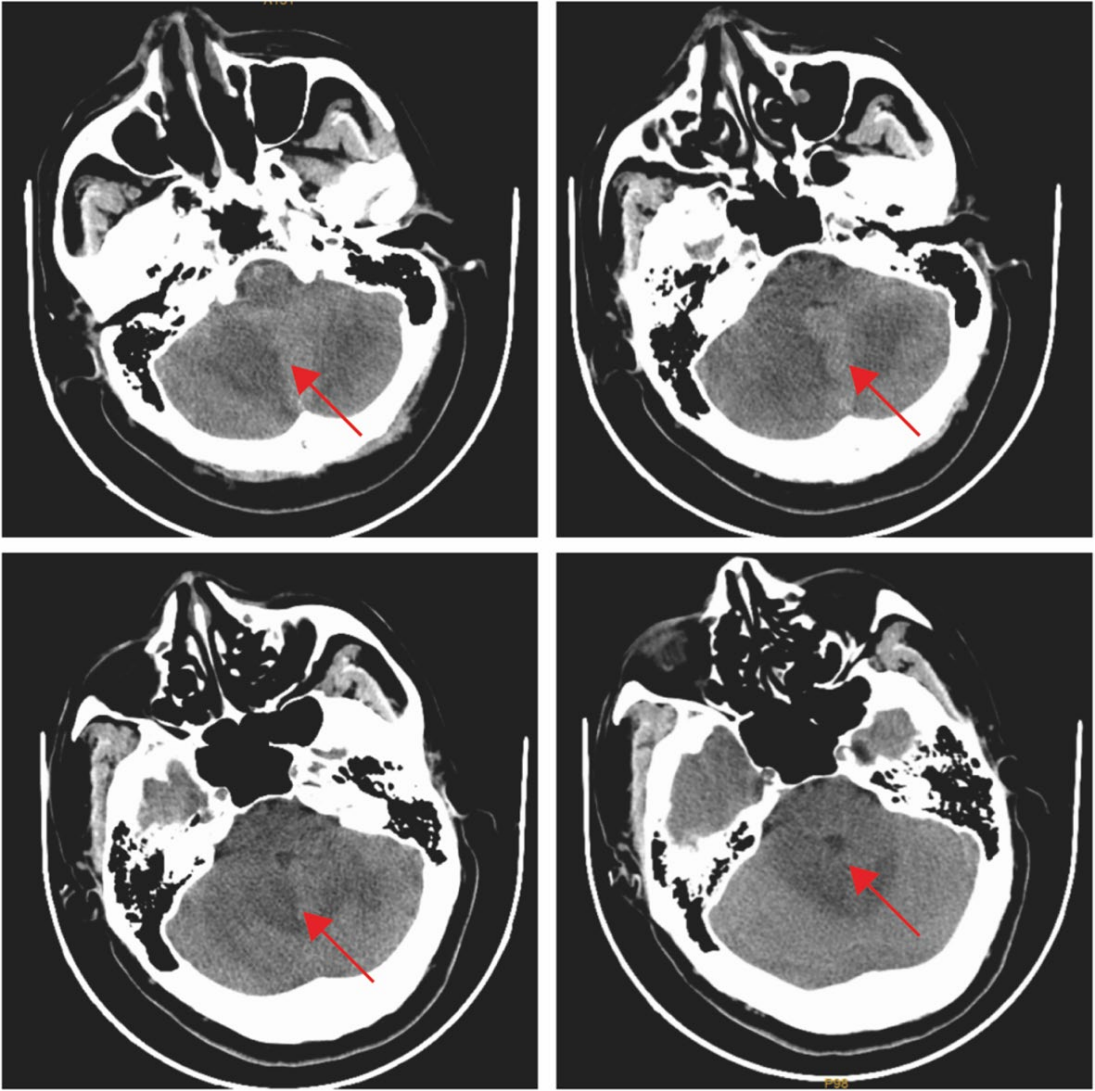
Plain head CT: bilateral cerebellar lesions. Arrowheads: infection lesions

**Fig. 2:**
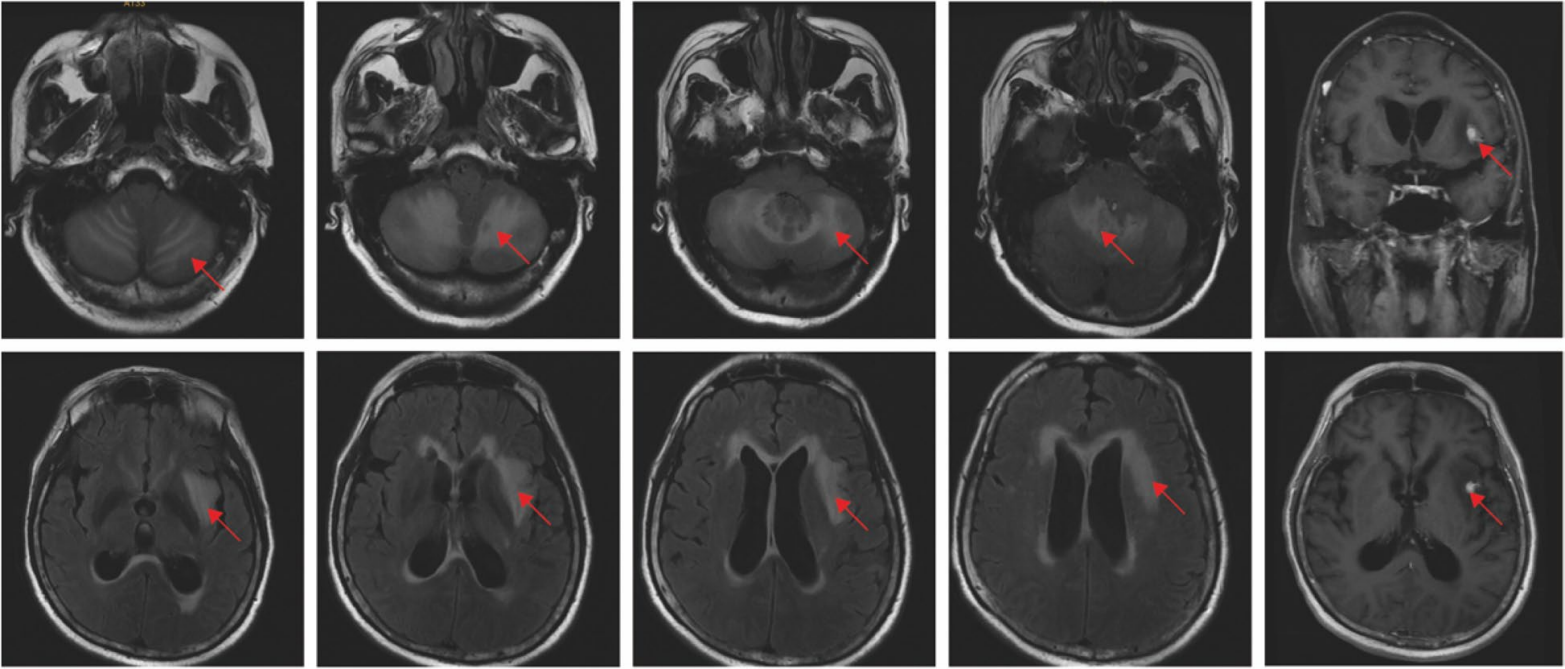
Plain and enhanced head MRI: abnormal signals in left lateral capsule, insular lobe and bilateral cerebellar hemispheres, with significant enhancement in the left insular nodule, left temporal nodule, and bilateral cerebellar meninges. Arrowheads: infection lesions

**Fig. 3:**
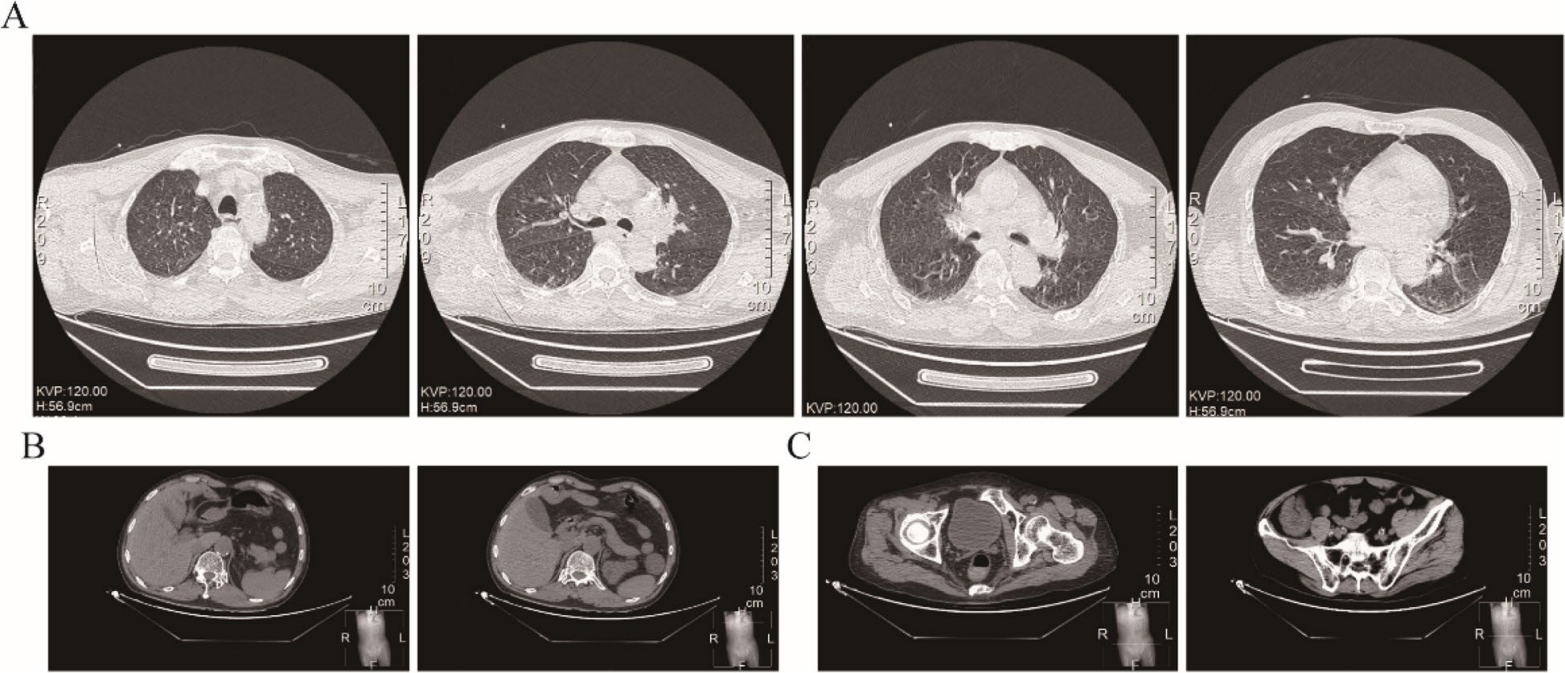
Chest and abdomen/pelvis CT: a small amount of pneumonia in both lower lung, hepatic cyst, renal cyst, prostatic hyperplasia

On the first day after admission, the patient had an abnormal body temperature of 38.2 °C (Day 1) and a deterioration in the level of consciousness. Examination of the nervous system showed drowsiness, whispering words, unwillingness to cooperate with the physical examination, equal and round bilateral pupils (3 mm in diameter) that were reactive to light, unwillingness to cooperate with the examination of limb muscle strength, deep and superficial sensibilities with a rough measurement result of grade 5, relatively poor stability of finger-to-nose test and heel-knee-tibia test, unwillingness to cooperate with the test for Romberg’s sign, negative pathological signs, stiff neck and four transverse finger breadths of the chin-sternum straight distance. The patient's head computed tomography(CT) showed bilateral cerebellar lesions and hydrocephalus (Fig. 1). Plain and enhanced magnetic resonance imaging(MRI) of the head showed abnormal signals in the bilateral cerebellar hemispheres and left basal ganglia-lateral capsule (Fig. 2). Meanwhile, to exclude malignancy-related immunocompromising conditions, tumor markers test, and chest/abdomen/pelvis CT were performed. All tumor markers were detected negative, which included CEA, AFP, CA153, CA199, PSA, CA72-4, CYFRA211, NSE, and SCC. Based on the patient's chest CT scan, a small amount of pneumonia was detected in the lower sections of both lungs **(**Fig. 3A). However, the sputum culture results showed negative bacterial infection, including actinomycetes. Abdomen/pelvis CT **(**Fig. 3B, C) indicated no infection in the abdominal cavity. The serological test also showed negative results for HIV, RPR, HBsAg, hepatitis B core antibody (anti-HBc), hepatitis B e antibody (anti-HBe), and HBe antigen. Thus, the patient received the ceftriaxone empirical therapy and blood culture at first.

However, the patient still had a recurrent fever, and the maximum body temperature was 39 °C, considering the possibility of intracranial infection. Therefore, lumbar puncture was performed based on mannitol dehydration to prevent cerebral hernia, with intracranial pressure of 115 mmH_2_O. Routine examination of the cerebrospinal fluid showed a white blood cell count of 0.62 × 10^9^/L, lymphocyte count of 85%, qualitative protein test result of 1 + , and activated monocyte percentage of 10%. Biochemical examination of the cerebrospinal fluid showed a protein level of 1371 mg/L without abnormality in glucose and chloride levels. Cryptococcus was undetected. There was no abnormality in the general bacterial smear, tuberculosis smear, and cerebrospinal fluid cytology. To make a definitive diagnosis, we collected cerebrospinal fluid for pathogenic metagenomics sequencing.

In the process of waiting for the pathogenic metagenomics sequencing results of cerebrospinal fluid, blood culture (Day 8) was positive for *actinomyces odontolyticus*, considering the possibility of *actinomyces odontolyticus* sepsis and intracranial *actinomyces odontolyticus* infection. Thus, penicillin treatment and continuous mannitol dehydration to reduce intracranial pressure was provided. The patient body temperature gradually dropped to normal levels(36.5 °C), but no improvement in symptoms of other intracranial infections.

**Fig. 4:**
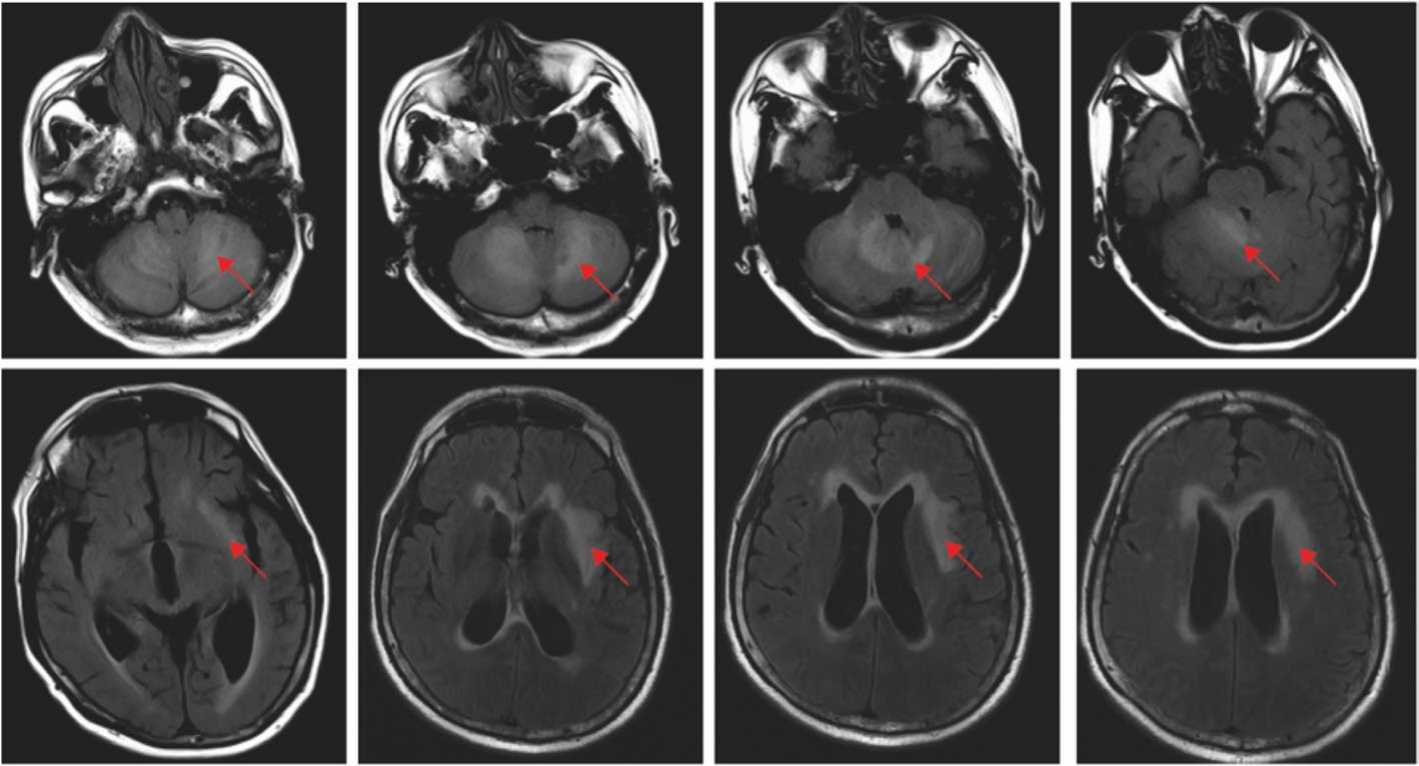
Plain head MRI: Abnormal signals in the left lateral capsule, insular lobe, and bilateral cerebellar hemispheres, with significant enhancement in the left insular nodule, left temporal nodule, and bilateral cerebellar meninges, with partial reduction of the focus than before. Arrowheads: infection lesions

**Fig. 5:**
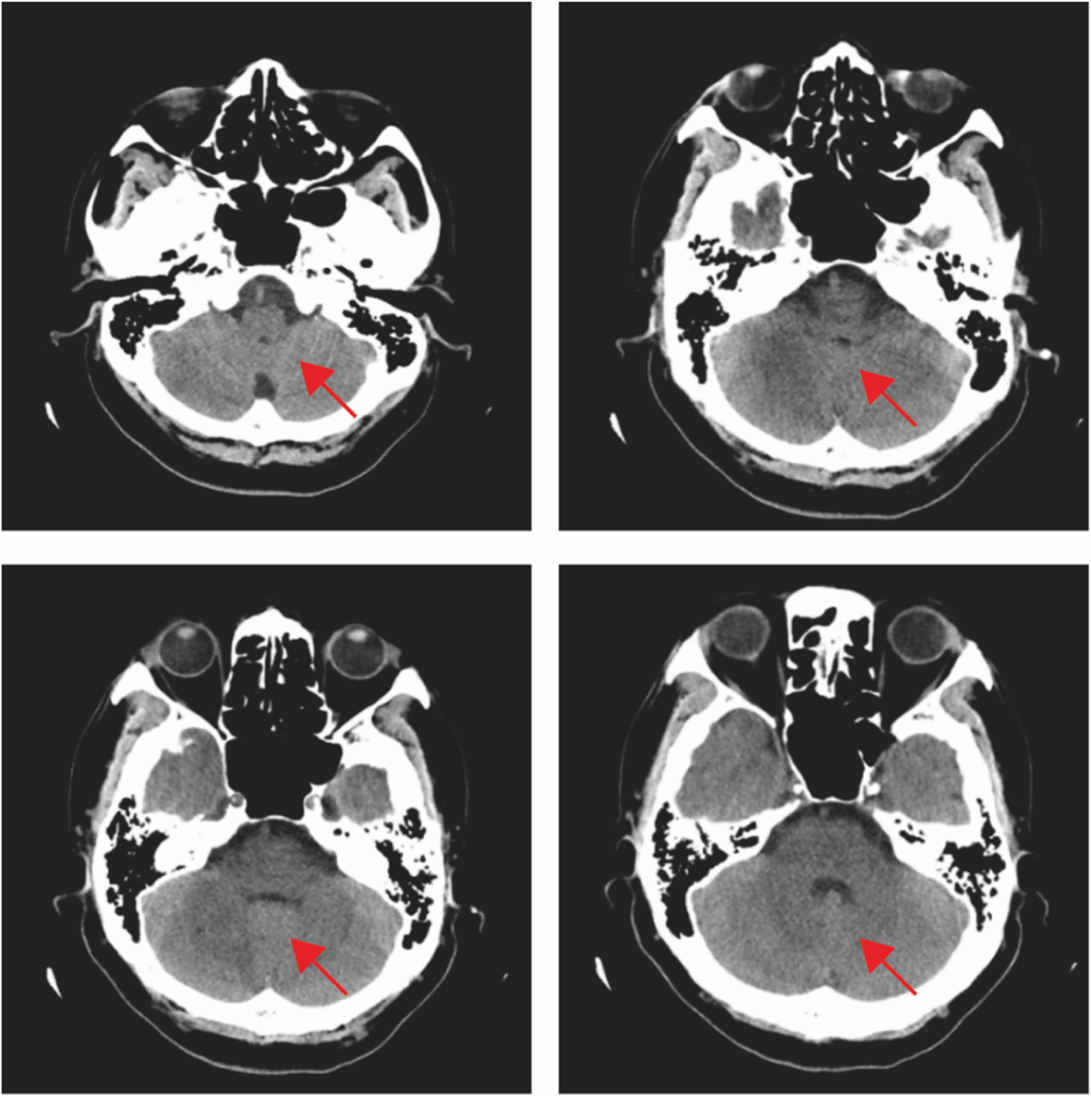
Plain head CT: bilateral cerebellar lesions were improved than before. Arrowheads: infection lesions

**Fig. 6:**
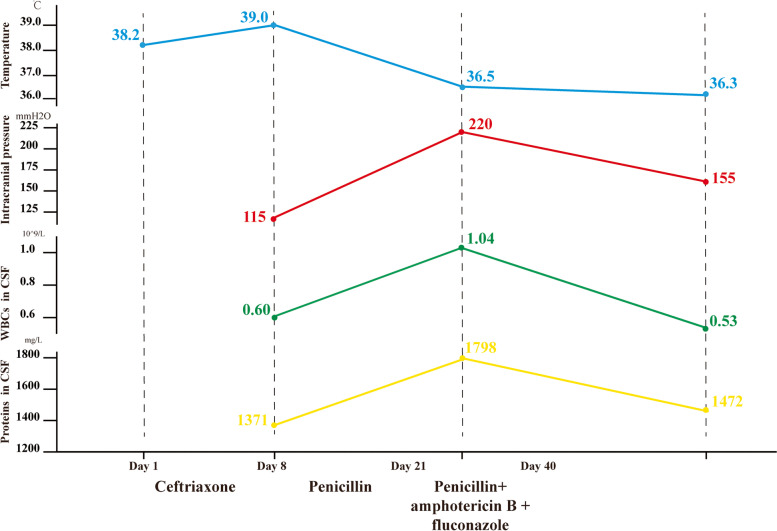
Patient's medication status, temperature changes, and cerebrospinal fluid test index. CSF, cerebrospinal fluid

During penicillin treatment, the gene examination of pathogenic microorganisms in the cerebrospinal fluid showed positive c*ryptococcus neoformans*, suggesting that the intracranial lesion would be ‘cryptococcal meningoencephalitis’ (Day 21). To further confirm the diagnosis, the patient underwent a re-examination of the lumbar puncture, with intracranial pressure of 220 mmH_2_O. A routine cerebrospinal fluid examination showed a white blood cell count of 1.04 × 10^9/^L, lymphocyte count of 76%, and qualitative protein test result 1 + . Biochemical examination of the cerebrospinal fluid showed a protein of 1798 mg/L, chloride level of 118.9 mmol/L, and glucose level of 1.98 mmol/L. But, cryptococcus was still undetected. There was also no abnormality on the general bacterial smear, tuberculosis smear, and cerebrospinal fluid cytology. Therefore, we re-examined the genome sequencing of pathogenic microorganisms, and the result still showed *cryptococcus neoformans* (positive). The serum cryptococcal antigen test kit (*Kangtai Biotechnology Co., LTD, China*) was used for cryptococcal antigen detection. This kit employs the double antibody sandwich method and includes negative and positive reference products as controls. The detection result showed (serum + cerebrospinal fluid) cryptococcal capsular polysaccharide antigen detection was positive (1: 80). Accordingly, ‘cryptococcal meningoencephalitis complicated with *actinomyces odontolyticus* sepsis’ was confirmed. For treatment, the patient was supplied with combined anti-infection therapy using ‘high-dose penicillin, liposome-encapsulated amphotericin B, and fluconazole.’ Subsequently, the patient did not develop fever again, with significant alleviation of headache. After 2 weeks, the patient was re-examined with a lumbar puncture (intracranial pressure of 155 mmH_2_O; routine examination of the cerebrospinal fluid showed white cell count of 0.53 × 10^9/^L, lymphocyte count of 100%; biochemical examination of the cerebrospinal fluid showed protein level of 1472 mg/L, chloride level of 121.7 mmol/L, and glucose level of 2.34 mmol/L), suggesting a reduction in cell count and protein. Moreover, a re-examination of plain and enhanced head MRI revealed a partial reduction of the intracranial abnormal enhanced focus (Figs. 4 and 5). The patient's medication status, temperature changes, and cerebrospinal fluid test index changes are shown in Fig. 6.

## Discussion and conclusions

‘Actinomycetes’ is generally used to describe gram-positive, non-spore-forming, and non-motile microorganisms that exhibit a pleomorphic morphology. As described above, actinomycosis is a chronic granulomatous disease with slow progression. In case of hypoxia and a decrease in local resistance caused by tissue injury or inflammation, it can cause endogenous infection involving various parts, such as the head and neck, chest and abdomen, pelvic cavity, and intracranial region. Cerebral actinomycetes are pretty rare and spread from superficial infection or actinomycosis in other parts. Besides, actinomycosis is commonly divided into localized brain abscesses and diffuse types [1–3]. Histopathological examination is the gold standard for diagnosing actinomycosis [4]. The first choice for routine treatment of actinomycosis is a high-dose penicillin intravenous drip (18 million–24 million U/day) for 2–6 weeks and sequential oral treatment for 6–12 months [2]. *Actinomyces odontolyticus* infection is a rare bacterial infection [5, 6]. In our case, routine anti-infection treatment was given after repeated fever, yet with poor therapeutic outcomes. A physical examination of the patient showed several adverse oral hygiene problems, such as ‘dental caries, broken teeth, and red and swollen gums’. Meanwhile, the result of the blood culture was positive for *actinomyces odontolyticus*, supporting the confirmed diagnosis of *actinomyces odontolyticus* sepsis. Simultaneously, plain and enhanced head MRI revealed abnormal signals in the left lateral capsule, insular lobe, and bilateral cerebellar hemispheres, with significant enhancement in the left insular nodule, left temporal nodule, and bilateral cerebellar meninges, considering the possibility of cerebral actinomycosis. However, the patient and his family refused further histopathological examination of the intracranial lesion, which restricted the diagnosis of cerebral actinomycosis.

As for the difference in this case, after the diagnosis of ‘*actinomyces odontolyticus* sepsis’, the patient showed partial improvement in the clinical symptoms after penicillin treatment. But there was still no significant improvement in the objective indexes during the re-examination of cerebrospinal fluid. Eventually, in accordance with the characteristics of brain MRI, the results of pathogenic metagenomics sequencing and cryptococcal capsular polysaccharide antigen detection in cerebrospinal fluid, the diagnosis of ‘cryptococcal meningoencephalitis complicated with *actinomyces odontolyticus* sepsis’ was confirmed. There would be a great possibility of delayed diagnosis in the patient when *cryptococcus neoformans* infection was not detected by pathogenic metagenomics sequencing of cerebrospinal fluid.

Clinically, cryptococcal meningoencephalitis in patients without HIV infection is a group of fungal meningitis with various clinical manifestations. Commonly, most patients may be chronically ill and may have symptoms for months before diagnosis. The common clinical manifestations are symptoms and signs of subacute or chronic meningoencephalitis, with fever observed in approximately 50% of patients [7]. In our case, the patient was a middle-aged male with no previous immune dysfunction diseases. The patient showed no abnormality in relevant indexes of HIV and rapid plasma reagin after admission and had a history of contact with poultry and livestock. The patient had subacute onset and persistent headache with fever, which was consistent with the characteristics of cryptococcal meningoencephalitis. In terms of the pathogenesis of non-HIV cerebral *cryptococcus neoformans* infection, the natural ability and evolution of pathogens may destroy and bypass the natural defense mechanisms of the hosts; in addition to its many virulence factors, *cryptococcus neoformans* showed high phenotypic plasticity and could escape host macrophages [8].

Through literature review, it was found that actinomycetes generally exist in combined infections, including Bacteroides, Clostridium, and Streptococcus, and rarely cause infection alone [9]. Two hypotheses concern the pathogenic mechanism of combined infection caused by actinomycetes. First, actinomycetes can grow well in a good anaerobic living environment created by other bacteria with the role of reducing oxygen pressure, both of which jointly promote and form actinomycosis. Second, actinomycetes and related bacteria survive in the form of aggregates, which can invade as a whole and play a role as an infected seed once there is a chance [10–12]. In this case, considering the patient's onset characteristics and contact history, it might suggest the existence of cryptococcal infection before admission. *actinomyces odontolyticus* sepsis was found during the hospitalization of the patient. As for the treatment, the patient was actively administered penicillin for anti-infection treatment, with improved clinical symptoms, suggesting the remission of sepsis. However, rather than improving, the cerebrospinal fluid markers of the patient highlighted the characteristics of cryptococcal infection of cerebrospinal fluid. In this regard, considering the diagnosis and treatment of the present case, it may suggest the necessity of monitoring the changes and progress of patients with fungal infection risk factors closely for those with unclear aetiology of infection clinically. In case of disease changes that the existing single pathogen infection cannot explain, it is necessary to complete additional detection items and pay attention to the possibility of combined infection. Once the combined infection is confirmed, it is important to apply specific drug interventions in time and detect the changes in indicators to improve the prognosis of patients in a timely manner.

Moreover, the patient underwent cerebrospinal fluid cytological examination, fungal culture, and India ink staining twice, without detection of *cryptococcus neoformans*. It may suggest the relatively low sensitivity of the above items. The patient was finally diagnosed with cryptococcal meningoencephalitis based on pathogenic metagenomics sequencing and cryptococcal capsular polysaccharide antigen detection in the serum and cerebrospinal fluid. Therefore, for unexplained intracranial infection, it is of great significance to adopt etiological test indicators with high specificity and sensitivity to improve the accuracy of clinical diagnosis and avoid missed diagnosis.

In conclusion, we first reported the combined infection of *actinomyces odontolyticus* sepsis and cryptococcal encephalitis. The summary of the diagnosis and treatment of this patient may improve clinicians’ understanding of this disease. It is important to conduct specific detection in time for suspicious cases and apply anti-infection therapy jointly in case of combined infection. This case report may inspire us to accumulate treatment experience and perform a comprehensive analysis to prevent missed diagnoses and misdiagnoses.

## Data Availability

Not applicable.

## Abbreviations

CT: Computed tomography
MRI: Magnetic resonance imaging
CSF: Cerebrospinal fluid
